# Metronomic 5-Fluorouracil and Vinorelbine Reduce Cancer Stemness and Modulate EZH2/NOTCH-1/STAT3 Signaling in Triple-Negative Breast Cancer Spheroids

**DOI:** 10.3390/ijms27010123

**Published:** 2025-12-22

**Authors:** Alice Ilari, Emanuela Grassilli, Mario Mauri, Marina E. Cazzaniga, Serena Capici, Marialuisa Lavitrano, Maria Grazia Cerrito

**Affiliations:** 1 School of Medicine and Surgery, Milano-Bicocca University, Via Cadore 48, 20900 Monza, Italy; a.ilari@campus.unimib.it (A.I.); mario.mauri@unimib.it (M.M.); marina.cazzaniga@unimib.it (M.E.C.); marialuisa.lavitrano@unimib.it (M.L.); 2 Department of Life Sciences, Health and Health Professions, Link Campus University, Via del Casale di San Pio V 44, 00165 Roma, Italy; e.grassilli@unilink.it; 3 Phase 1 Research Centre, Fondazione IRCCS San Gerardo dei Tintori, Via Pergolesi 33, 20900 Monza, Italy; serena.capici@irccs-sangerardo.it

**Keywords:** Triple-Negative Breast Cancer (TNBC), metronomic therapy (mCHT), cancer stem cells (CSCs), stemness markers, EZH2, NOTCH-1, STAT3

## Abstract

Triple Negative Breast Cancers (TNBCs) are heterogeneous and aggressive tumors with a median overall survival of less than two years. Despite the availability of new drugs, the prognosis remains poor, implicating a more aggressive clinical course in the metastatic setting. This study investigated the effects of metronomic treatment (mCHT) with 5-fluorouracil (5-FU) plus vinorelbine (VNR) on spheroids derived from two different TNBC cell lines (BT-549 and MDA-MB-231) and a patient-derived primary cell line (MS-186). mCHT significantly reduced spheroid growth and altered spheroid architecture, with a pronounced effect in second-generation spheroids, enriched in self-renewing cancer stem cells (CSCs). Expression of CSC-related markers (CD44, CD133, NOTCH-1, and MYC) was more significantly altered—both at the mRNA and protein levels—by mCHT than by standard treatment (STD). In MS-186-derived spheroids, mCHT downregulated EZH2 and STAT3, key regulators of CSC maintenance, and reduced H3K27ac, suggesting a global epigenetic reprogramming. Unlike STD, which partially and transiently reduced stemness markers, mCHT achieved sustained suppression, indicating preferential targeting of therapy-resistant CSCs. These results indicate mCHT as a promising strategy for specifically aiming at the CSC-like compartment in TNBC, underscoring a therapeutic approach that reprograms key epigenetic networks and overcomes resistance to treatment.

## 1. Introduction

Triple-negative breast cancer (TNBC) is one of the most aggressive and therapeutically challenging breast cancer subtypes, characterized by the absence of the estrogen receptor, progesterone receptor, and human epidermal growth factor receptor 2 expression [1,2]. This molecular profile results in a lack of targeted treatment options, leaving cytotoxic chemotherapy as the mainstay of treatment for most patients. Although many patients initially respond to maximum tolerated dose (MTD) chemotherapy, relapse is common and often with an aggressive and treatment-resistant phenotype, resulting in shorter disease-free survival and overall survival [3]. MTD—which in our experimental design corresponds to the STD treatment—mainly acts by inducing cytotoxicity in cancer cells, preferentially targeting the bulk of rapidly proliferating cells. This selective elimination process often fails to eradicate the quiescent CSC subpopulation, instead acting as a strong selective pressure that favors the enrichment of highly resistant cells and fosters aggressive tumor recurrence. CSCs are characterized by their capacity for self-renewal, tumor initiation, and resistance to therapy and are considered to contribute significantly to TNBC progression and recurrence [4]. The ability of CSCs to resist conventional therapies makes them a key target for the development of anticancer treatments aimed specifically at this subpopulation. Despite growing evidence implicating CSCs in the aggressiveness of TNBC, their contribution to key signaling and epigenetic pathways remains underexplored.

Recently, several promising therapeutic options have been developed for TNBC. Immune checkpoint inhibitors such as atezolizumab and pembrolizumab have shown improved outcomes in patients with PD-L1-positive TNBC when combined with chemotherapy [5]. Another significant advancement is sacituzumab govitecan, an antibody–drug conjugate targeting the cell surface antigen Trop-2, which has shown significant clinical benefit even in heavily pretreated metastatic TNBC patients. This therapy combines the specificity of a Trop-2-targeted monoclonal antibody with the cytotoxic activity of a topoisomerase I inhibitor, demonstrating clinical benefits even in advanced cases [6,7]. Although these two examples represent important progress, especially in metastatic TNBC patients, the aggressive nature of TNBC, its early metastasis spread, and elevated intratumoral heterogeneity highlight the urgent need for identification and development of additional innovative therapeutic strategies. One promising strategy to address this challenge is metronomic chemotherapy (mCHT). This approach involves the administration of low doses of chemotherapy at regular intervals with minimal drug-free breaks. Unlike maximum tolerated dose regimens, which mainly act by inducing cancer cell cytotoxicity, mCHT exerts its effects by targeting different tumoral aspects. We previously evaluated the combination of Capecitabine (the oral prodrug of 5-fluorouracil, 5-FU) and Vinorelbine (VNR) in mCHT regimen in breast cancer patients, demonstrating a disease control rate and manageable toxicity profiles [8]. Our previous preclinical studies investigated the 5-FU+VNR combination and showed that mCHT administration was significantly more effective than the STD schedule, with superior inhibitory effect on cell growth and on the activation of apoptosis and autophagy mechanisms in TNBC cell lines MDA-MB-231 and BT-549 [9]. Later, our preclinical investigations confirmed the multitarget action of mCHT, revealing a direct anti-tumor effect, anti-angiogenic properties, and inhibition of TNBC cell migration/invasion, via downregulation of the FAK/VEGFR2/VEGF axis [10]. This pleiotropic modality of action reduces systemic toxicity and may affect therapy-resistant cell populations, including CSCs [11]. However, the application of mCHT in TNBC, particularly its effects on CSC-related pathways and epigenetic regulation, remains underexplored.

The present study extends these findings by providing molecular evidence that mCHT administration of 5-FU+VNR inhibits spheroid formation and stemness-related gene expression across commercial TNBC cell lines (BT-549 and MDA-MB-231) and a TNBC patient-derived cell line (MS-186) [12], compared to conventional STD treatment. The spheroid formation assay provides a robust in vitro system to evaluate CSC activity, as it closely recapitulates the three-dimensional architecture and microenvironment of tumors compared to traditional two-dimensional cultures [13]. To evaluate sustained treatment effects, we used first and second (1st and 2nd) generation spheroids to analyze the long-term effects of mCHT vs. standard STD treatment on the key markers of stemness such as CD44, CD133, MYC, and NOTCH-1. These markers play pivotal roles in maintaining the CSC phenotype and have been associated with treatment resistance and metastatic potential [14]. Our results show that, in commercial cell line-derived spheroids, both STD and mCHT regimens modulate the expression of these markers, albeit somewhat differently, likely due to the different genetic backgrounds of the cell lines. However, results are clear-cut when using spheroids obtained from the primary cell line MS-186: in this case, 5-FU+VNR given according to the mCHT, but not the STD schedule, abolished the expression of all CSC markers tested—both at the RNA and protein levels—in 2nd generation spheroids. Mechanistically, mCHT in this setting induces marked downregulation of enhancer of zeste homolog 2 (EZH2) and H3K27ac (an active enhancer mark) and reduces STAT3 expression and phosphorylation.

To our knowledge, this is the first study to demonstrate that long-term mCHT 5-FU+VNR induces persistent CSC depletion and causes epigenetic reprogramming in TNBC spheroids, supporting its potential as a therapeutic strategy for this aggressive breast cancer subtype.

## 2. Results

### 2.1. mCHT Reduces the Growth of TNBC Cell Line-Derived Spheroids

**Figure 1:**
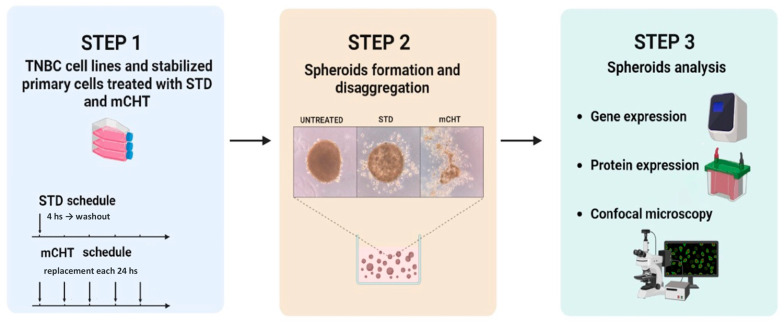
Experimental design for evaluating the effects of mCHT treatment on TNBC stemness. Both TNBC commercial and stabilized primary cell lines were treated with STD and mCHT schedules of 5-FU+VNR, as detailed in the Section 4. Surviving cells were used to generate 1st-generation 3D spheroids, which were disaggregated after 10 days to form 2nd-generation spheroids. After another 10 days, cells derived from the 1st generation of spheroids were analyzed for stemness-associated gene and protein expression using qRT-PCR, Western blotting, and confocal microscopy.

To assess the effect of mCHT treatment on a surrogate model for studying stem-like properties in vitro, we analyzed spheroids formed after STD or mCHT treatment of BT-549 and MDA-MB-231 TNBC cell lines (Figure 1).

**Figure 2:**
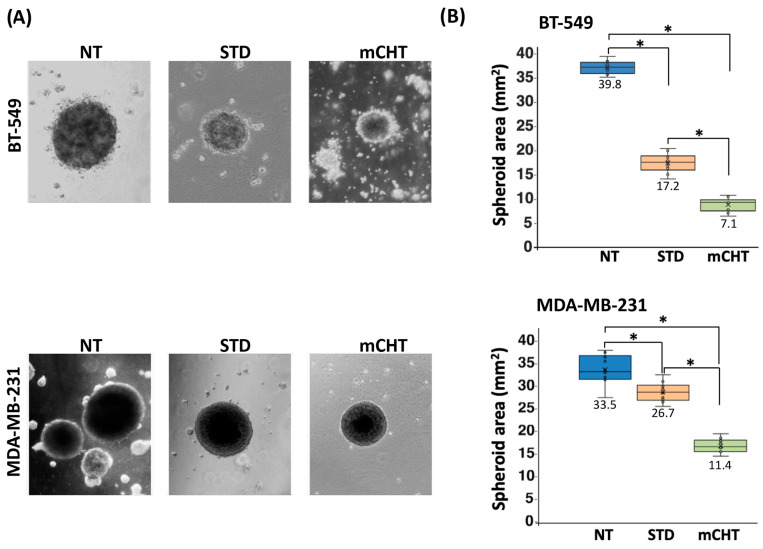
Effect of mCHT and STD chemotherapy on the growth of TNBC cell line-derived spheroids. (**A**) Representative images of BT-549 and MDA-MB-231 cell-derived spheroids grown after STD or mCHT treatment with 5-FU+VNR. Spheroids derived from cells treated with vehicle alone (NT) are shown as controls. The average area of the spheroids is expressed in mm^2^ and is calculated one week after the end of treatments and the reseeding of surviving cells in Matrigel. (**B**) Box plots on the right show the quantification of the area (mm^2^) of BT-549 (top panel) and MDA-MB-231 (bottom panel) spheroids subjected to NT, STD, and mCHT treatments. For each treatment, except for the mCHT treatment (where very few spheroids formed), 15 spheroids were evaluated from each of three independent experiments. Statistical analysis was performed using a two-tailed *T* test. Asterisks indicate *p* < 0.05. Numbers and the symbol (×) indicate the mean area (mm^2^) of the spheroids, while the horizontal line indicates the median.

Surviving cells were cultured in Matrigel to allow spheroid formation and after one week the surface areas of the spheroids were measured (Figure 2).

For both cell lines, the average surface area of the spheroids formed after mCHT treatment is severely reduced compared to the area of those grown after vehicle alone (NT) and STD treatments. In addition, when compared to spheroids derived from STD-treated or NT cells, spheroids derived from mCHT-treated cells are also fewer and generally less compact. Notably, spheroids from mCHT-treated cells are loosely organized and less viable, indicating a significant disruption of self-renewal ability.

Overall, our data suggest that low-dose continuous exposure is more effective at limiting tumor cell growth than a single high dose.

### 2.2. mCHT Treatment Impairs the Formation of 2nd-Generation Spheroids Derived from TNBC Cell Lines

**Figure 3:**
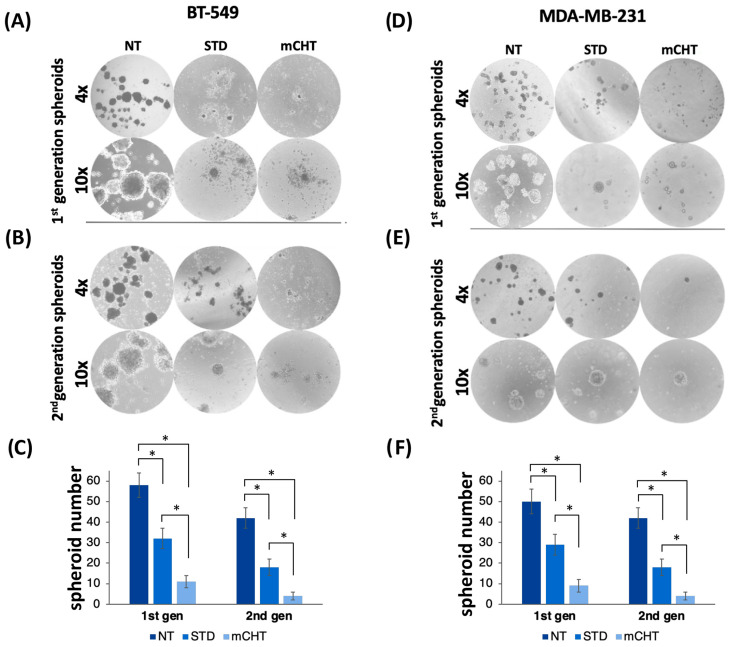
mCHT is more efficient than STD in impairing the formation of 1st- and 2nd-generation spheroids derived from TNBC cell lines. 1st-generation spheroids derived as described in Materials and Methods from BT-549 (**A**) and MDA-MB-231 (**D**) and treated under the STD or mCHT schedule. Cells treated with vehicle alone are indicated as NT. 2nd-generation spheroids (**B**,**E**) were obtained by replating the surviving cells after disaggregation of 1st-generation spheroids. Images were taken 10 days after replating, as described in the Section 4. (**C**,**F**) Quantification of 1st- and 2nd-generation BT-549- and MDA-MB-231-derived spheroid number under NT, STD, and mCHT treatments. Statistical analysis was performed using two-tailed *T* test. Values represent mean ± SEM of three experiments. Asterisks indicate *p* < 0.05.

Following the observation that mCHT strongly reduces the growth of 1st-generation spheroids, we next extended the evaluation to the growth capacity of 2nd-generation spheroids, which were obtained after disaggregating and reseeding cells from the 1st-generation spheroids. Compared to 1st-generation spheroids (Figure 3A,C), the inhibitory effect is even more pronounced in 2nd-generation spheroids (Figure 3B–D).

Spheroids derived from mCHT-treated cells show a dramatic reduction in their ability to organize a three-dimensional structure. In contrast, spheroids derived from STD-treated cells remain relatively numerous and more structured, indicating a persistence of self-renewal ability.

These findings suggest that 5-FU+VNR administered on a mCHT schedule exerts a more sustained inhibitory effect on TNBC spheroid formation than STD administration, likely impairing survival, propagation, and self-renewal potential, indicating its potential in targeting the stem-like subpopulation.

### 2.3. mCHT Treatment Reduces the Expression of Stemness-Associated Genes in TNBC Spheroids

**Figure 4:**
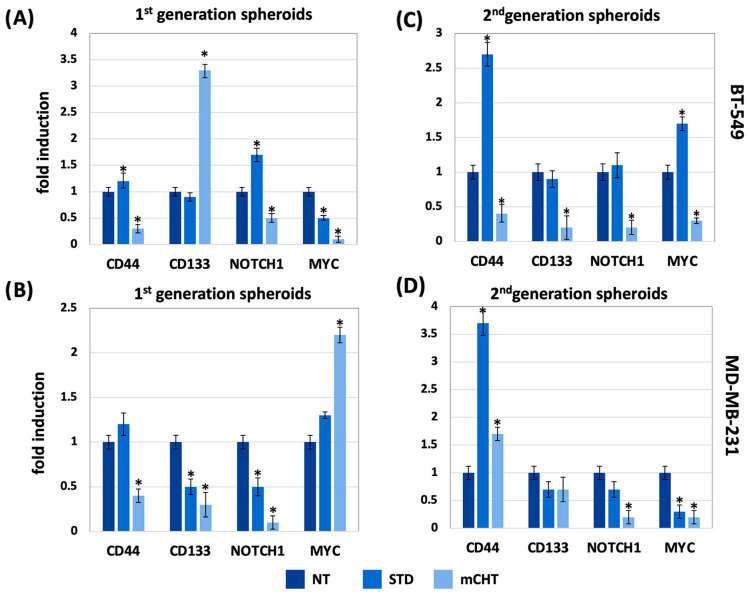
mCHT treatment suppresses expression of stemness-related genes in TNBC cell line-derived spheroids. RNA was extracted from BT-549 (**A**) and MDA-MB-231 (**B**) surviving cells recovered from the culture of 1st- and 2nd-generation spheroids treated with vehicle alone (NT) or treated with either STD or mCHT 5-FU+VNR schedule, as described in Section 4. 1st-generation spheroids are shown in (**A**,**C**), and 2nd-generation spheroids are shown in (**B**,**D**). Expression levels of stemness-related genes (CD44, CD133, NOTCH-1, and MYC) were normalized to GAPDH and reported as relative fold changes. Statistical analysis was performed using two-tailed *T* test. Values represent mean ± SEM of three separate experiments. Asterisks indicate *p* < 0.05 between NT and mCHT- or STD-treated cells.

To investigate the impact of mCHT treatment on the expression of stemness-associated genes, we performed qRT-PCR analysis on 1st- and 2nd-generation spheroids derived from BT-549 and MDA-MB-231 cells following treatment with either an STD or mCHT schedule combining 5-FU+VNR. Overall, when examining the expression of CD44, CD133, NOTCH-1, and MYC, we found that mCHT treatment more strongly reduces their mRNA levels than STD regimen. In particular, a significant downregulation is observed in 1st-generation spheroids (Figure 4A,C), except for CD133 in BT-549 and MYC in MDA-MB-231.

The reduction is even more pronounced in 2nd-generation spheroids (Figure 4B,D), indicating a sustained anti-stemness effect of the mCHT regimen. Notably, the reduction in CD133 and MYC expression is particularly strong in mCHT-treated spheroids of BT-549 and MDA-MB-231, respectively. These results suggest that the administration of 5-FU combined with VNR under mCHT schedule represents a more effective strategy than STD treatment in impairing stem-like properties in TNBC cells.

### 2.4. mCHT Is More Effective than STD Schedule in Decreasing Stemness-Related Protein Levels in Spheroids Derived from TNBC Commercial Cell Lines

**Figure 5:**
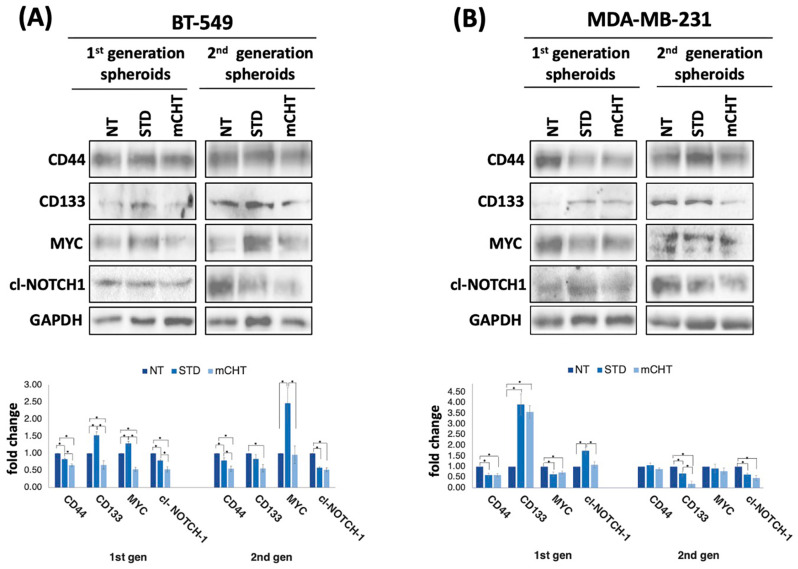
mCHT is more effective than STD schedule in decreasing stemness-related gene expression in both in 1st- and 2nd-generation spheroids derived from TNBC commercial cell lines. ((**A**,**B**) **Top**) Representative Western blots (from a single experiment) using proteins extracted from BT-549 (**A**) and MDA-MB-231 (**B**) surviving cells recovered from the culture of 1st- and 2nd-generation spheroids at the end of STD and mCHT treatments. NT indicates lysates from cells treated with vehicle alone. cl-NOTCH-1 indicates the cleaved form of NOTCH-1 that corresponds to the transcriptionally active intracellular domain of NOTCH-1. GAPDH was used as a loading control and to perform densitometric evaluation of relative protein levels. ((**A**,**B**) **Bottom**). Graphs plotting the densitometric quantitation of protein levels reported relative to NT (set to 1). Statistical analysis was performed using two-tailed *T* test. Values represent mean ± SEM of three experiments. Asterisks indicate *p* < 0.05.

To confirm the differential effects of mCHT and STD treatments at the protein level, we performed Western blot analysis on lysates from 1st- and 2nd-generation spheroids derived from BT-549 and MDA-MB-231 cells upon STD versus mCHT administration of 5-FU+VNR (Figure 5A,B).

Spheroids treated with vehicle alone (NT) display variable levels of the stemness-related markers CD44, CD133, NOTCH-1, and MYC, which are differently modulated by the two schedules of treatment. CD44 expression levels are more significantly reduced by mCHT than by STD treatment in both 1st-generation and 2nd-generation spheroids derived from BT-549 cells. In contrast, they are equally affected in MDA-MB-231-derived 1st-generation spheroids, whereas they are constant 2nd-generation spheroids. CD133 is strongly induced by both mCHT and STD in 1st-generation spheroids derived from MD-MB-231 but is only depleted by mCHT schedule in 2nd-generation spheroids. In 1st-generation BT-549-derived spheroids, CD133 levels are increased by STD treatment and decreased by mCHT but are only mildly affected by both schedules of treatment in 2nd-generation spheroids. Notably, in the same spheroids, MYC levels are decreased by mCHT schedule and increased in response to STD treatment, with the induction particularly strong in 2nd-generation spheroids; in contrast, in MDA-MB-231-derived spheroids, MYC expression decreases following both types of treatment in 1st-generation spheroids, whereas it is not significantly affected in 2nd-generation spheroids. The cleaved (and therefore transcriptionally active) form of NOTCH-1 is more significantly reduced by mCHT than by STD treatment in BT-549-derived 1st-generation spheroids, whilst it is increased by STD schedule in MDA-MB-231-derived 1st-generation spheroids. At variance, cleaved NOTCH-1 levels are reduced by both types of treatments in 2nd-generation spheroids derived from both cell lines.

Altogether, the expression of stemness-related genes is heterogeneously affected by the two different schedules of treatments, with a reduction in their expression prevalent under the mCHT schedule. Instead, gene expression variation is milder in spheroids derived from STD-treated cells.

### 2.5. TNBC Cell Lines-Derived Spheroids Treated with mCHT Schedule Are Characterized by Depletion of Stemness Markers and Cytoskeletal Disruption

**Figure 6:**
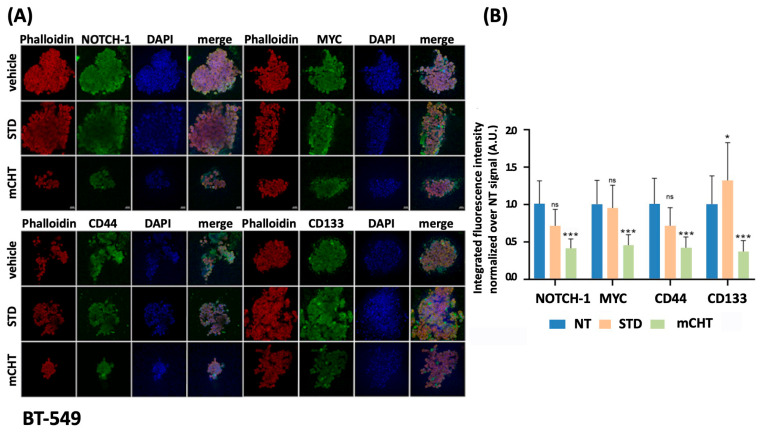
mCHT reduces BT-549-derived spheroid’s size and stemness markers expression. (**A**) Immunofluorescence analysis of spheroids derived from BT-549 treated with vehicle alone (NT) or following STD or mCHT administration of 5-FU+VNR. Spheroids were stained for stemness markers (green), phalloidin to stain cytoskeleton F-actin (red), and DAPI to stain nucleus (blue). Composite images show merged channels. (**B**) Histograms show the integrated fluorescence intensity quantification normalized over vehicle alone condition. (* *p* < 0.05; *** *p*< 0.001; ns: *p* is not significant). Scale bar: 20 μM.

**Figure 7:**
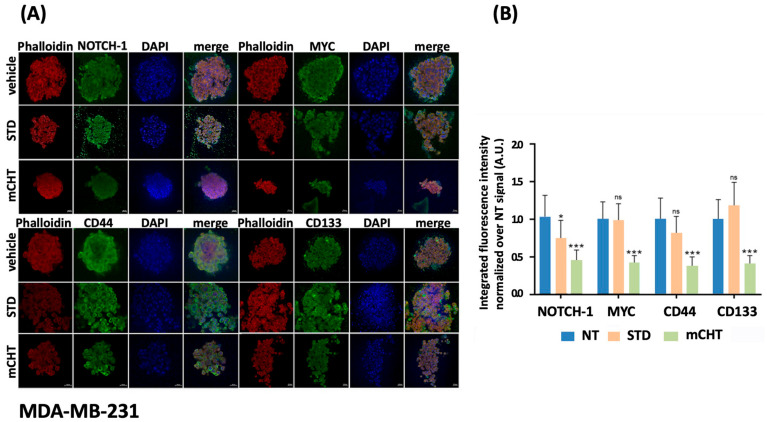
mCHT reduces MDA-MB-231-derived spheroid’s size and stemness markers expression. (**A**) Immunofluorescence analysis of spheroids derived from MDA-MB-231 treated with vehicle alone (NT) or following STD or mCHT administration of 5-FU+VNR. Spheroids were stained for stemness markers (green), phalloidin to stain cytoskeleton F-actin (red), and DAPI to stain nucleus (blue). Composite images show merged channels. (**B**) Histograms show the integrated fluorescence intensity quantification normalized over vehicle alone condition (* *p* < 0.05; *** *p*< 0.001; ns: *p* is not significant). Scale bar: 20 μM.

To localize the expression of stemness markers within the 3D tumor model, we performed immunofluorescence analysis on BT-549 and MDA-MB-231 spheroids formed from cells treated with STD or mCHT schedule of 5-FU+VNR (Figure 6 and Figure 7). NT spheroids show elevated NOTCH-1 and MYC fluorescence signals, indicating elevated expression levels. In BT-549-derived spheroids, a reduction in the intensity of these signals is observed upon the STD treatment, although they remain detectable (Figure 6A).

Notably, NOTCH-1 signal is even more pronounced in the MDA-MB-231-derived spheroids (Figure 7A,B); mCHT administration results in a drastic reduction or near absence of both markers, suggesting a strong downregulation in spheroids derived from both cell lines. CD44 and CD133 exhibit moderate to high signal intensity in untreated spheroids derived from both cell lines (Figure 6A,B and Figure 7A,B).

Interestingly, CD133 signal is increased in both types of spheroids upon SDT treatment, possibly indicating the selective survival of a subpopulation (Figure 6A,B and Figure 7A,B).

Under the mCHT schedule, both markers show very low to undetectable fluorescence, indicating effective depletion of stem-like cells in spheroid derived from both cell lines. The phalloidin signal is intense and distributed in NT and STD-treated spheroids, outlining intact actin structures. In contrast, upon mCHT schedule, phalloidin signal is significantly reduced, showing a small size and alterations in cytoskeleton structure (Figure 6A,B and Figure 7A,B). These changes, which indicate a loss of structural integrity, can affect cellular properties such as adhesion, migration, and invasiveness, potentially leading to a reduction in cancer aggressiveness.

### 2.6. mCHT vs. STD Schedule of 5-FU+VNR Significantly Enhances Cytotoxicity in Patient-Derived Primary MS-186 Cell Line

To better represent the complexity and heterogeneity of patient tumors, we used MS-186 cells, an established primary culture derived from a TNBC patient [11]. This model maintains features of the original tumor, including stem cell-like properties and treatment resistance, making it a clinically relevant system for studying treatment response and stemness-related mechanisms in TNBC.

First, we performed cytotoxicity assays, as detailed in Section 4, which show that mCHT administration dramatically enhances drug effectiveness compared to the STD schedule (Table 1).

Specifically, the IC_50_ of 5-FU given mCHT is 24 times lower than given STD (26,000 nM vs. 625,000 nM). Similarly, the IC_50_ of VNR is 15 times lower in the mCHT vs. STD schedule (4 nM vs. 60 nM). Combination indexes (CI) analysis, calculated using the Chou–Talalay method [15] of the IC_50_ values, confirmed an additive effect in both regimens (CI ≈ 1.0), suggesting that mCHT not only reduces the effective drug concentration required for cytotoxicity but does so, apparently, without compromising the drug–drug interaction profile, sustaining its potential clinical advantage in reducing systemic toxicity while maintaining therapeutic efficacy.

### 2.7. mCHT Reduces the Ability to Form 1st-Generation Spheroids and the Expression of Stemness Markers in Patient-Derived Primary MS-186 Cell Line

**Figure 8:**
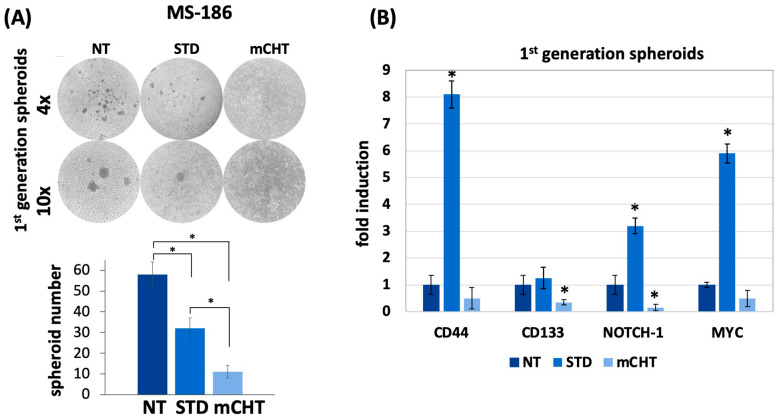
mCHT reduces the ability to form 1st-generation spheroids and the expression of stemness markers in MS-186 cells ((**A**) **Top**) 1st-generation spheroids derived as described in Section 4 were treated with vehicle alone (NT) or under the STD or mCHT schedule. ((**A**) **Bottom**) quantification of spheroid area (mm^2^) in MS-186 1st-generation spheroids under NT, STD, and mCHT treatment (10× magnification) For each treatment, but for mCHT (where very few spheroids were formed), 10 spheroids were evaluated. Statistical analysis was performed using two-tailed *T* test. Values represent mean ± SEM of three experiments. Asterisks indicate *p* < 0.05. (**B**) Relative gene expression analysis (RT-qPCR) of the stemness-associated markers CD44, CD133, NOTCH-1, and MYC. Statistical analysis was performed using two-tailed *T* test. Values represent mean ± SEM of three experiments. Asterisks indicate *p* < 0.05 between NT and mCHT- or STD-treated cells.

MS186 cells were used to generate 3D spheroids following treatment with STD or mCHT regimens of 5-FU+VNR. Brightfield images after 7 days revealed that mCHT treatment markedly impaired spheroid formation, producing smaller and less compact structures compared to both NT and STD conditions (Figure 8A).

Gene expression analysis by RT-qPCR reveals a substantial downregulation of stemness-related markers (CD44, CD133, NOTCH-1, and MYC) in spheroids derived from MS-186 cells treated with mCHT (Figure 8B), supporting the significant reduction in proliferative capacity and self-renewal observed in Figure 8A. Interestingly, spheroids derived from MS-186 cells treated with STD show an opposite pattern of expression with all genes but one (CD113) strongly upregulated, likely indicative of stemness-associated drug resistance.

Altogether, these results indicate that 5-FU+VNR given mCHT disrupts spheroid integrity and downregulates the expression of stemness-related genes in a patient-derived primary cell line, showing the potential to target resistant and stem-cell-like tumor cell populations more efficiently than STD treatment.

### 2.8. mCHT Reduces Stemness-Related Protein Expression in 1st- and 2nd-Generation Spheroids Derived from MS-186 Primary TNBC Cells

**Figure 9:**
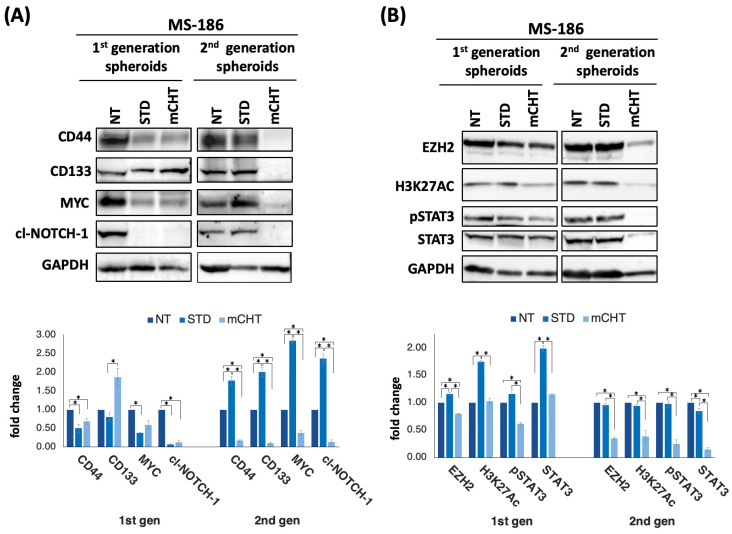
mCHT downregulates stemness-associated protein and epigenetic regulators in 1st- and 2nd-generation spheroids derived from MS-186 cells. ((**A**,**B**), **Top**) Representative Western blots using proteins extracted from MS-186-derived 1st- and 2nd-generation surviving spheroids recovered at the end of STD and mCHT treatments. Cl-NOTCH-1 indicates the cleaved form of NOTCH-1 that corresponds to the intracellular domain of NOTCH-1. NT indicates lysates from cells treated with vehicle alone. GAPDH was used as a loading control and to perform densitometric evaluation of relative protein levels. ((**A**,**B**), **Bottom**). Graphs plotting the densitometric quantitation of protein levels reported relative to NT (set to 1). Statistical analysis was performed using two-tailed *T* test. Values represent mean ± SEM of three experiments. Asterisks indicate *p* < 0.05.

To evaluate the molecular impact of mCHT on MS-186 cells, we analyzed protein expression in 1st- and 2nd-generation spheroids derived from cells seeded and grown after treatment with 5-FU+VNR under STD or mCHT schedules. Western blot analysis of lysates derived from 1st-generation spheroids shows that both STD and mCHT treatment significantly reduces the expression of all stemness-associated genes but CD133, which is upregulated by mCHT treatment (Figure 9A).

**Figure 10:**
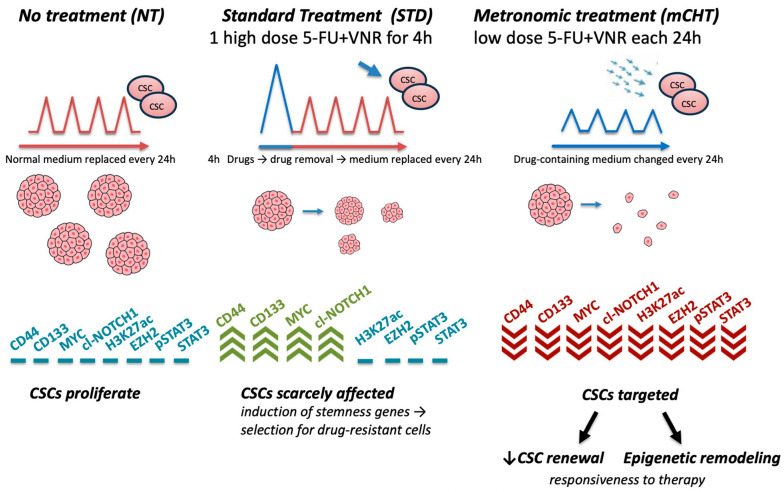
Summary of the results obtained on 2nd-generation spheroids derived from primary TNBC cell line MS-186, disaggregated from 1st-generation spheroids after STD or mCHT treatments. In the absence of treatment (NT), CSCs continue to proliferate and maintain stemness markers (CD44, CD133, MYC, cleaved NOTCH-1), epigenetic regulators (H3K27ac, EZH2), and signaling molecules (p-STAT3/STAT3). The STD treatment partly reduces the mass and the number of spheroids but induces high levels of stemness markers, which continue to activate stemness pathways and sustain drug resistance. In contrast, mCHT dramatically impairs spheroid formation, epigenetic signaling, and associated markers, thereby improving treatment responses.

However, in the 2nd-generation spheroids, all genes are upregulated under STD treatment, whereas mCHT completely suppresses all proteins tested (Figure 9A). Interestingly, EZH2 and its downstream target STAT3/pSTAT3 are increased by STD treatment in 1st-generation spheroids and return to basal levels in 2nd-generation spheroids, whereas they are decreased by mCHT schedule in both types of spheroids. Notably, the decrease is even more remarkable in 2nd-generation spheroids. In addition, levels of H3K27ac—a marker of transcriptionally active enhancers, ref. [16]—are also upregulated by STD treatment, whereas they are strongly reduced by mCHT (Figure 9B), suggesting that mCHT reshapes the chromatin landscape and enforces a sustained inhibition of stemness-related transcriptional programs (as summarized in Figure 10).

Altogether, these findings suggest that mCHT not only suppresses CSC-associated markers more efficiently than STD but also drives epigenetic changes that may be involved in TNBC spheroid growth and self-renewal.

## 3. Discussion

TNBC remains one of the most challenging breast cancer subtypes to treat, mainly due to its intrinsic cellular heterogeneity, the absence of key actionable endocrine or HER2 targets, and a propensity for early recurrence and metastasis. TNBC is an aggressive neoplasia with a median overall survival of less than two years, underlining the limitations of current treatments [3]. STD, high-dose regimens preferentially target rapidly dividing tumor cells, whereas the quiescent CSC fraction frequently escapes treatment. This incomplete clearance imposes a strong selective pressure that promotes the survival and expansion of resistant clones, driving aggressive tumor recurrences and contributing to therapeutic failure. mCHT, instead, with its multimodal mechanism of action, successfully targets the CSC population while preserving the key advantages of this approach, i.e., easy administration, low toxicity, and multimodal mechanism of action. All these factors have significantly contributed to the common use of mCHT in several other types of cancer, such as NSCLC, gastrointestinal, gynecological, and nasopharyngeal cancers [17]. CSCs are highly plastic subpopulations of cells characterized by tumor-initiating capacity and intrinsic resistance to cytotoxic treatments [18]. Effective, long-term therapeutic strategies must therefore focus on agents capable of eliminating or functionally reprogramming the CSC compartment.

In this study, we demonstrate that the efficacy of the 5-FU+VNR combination is critically dependent on the administration schedule. The mCHT treatment exerts a more profound inhibitory effect on spheroid formation and stemness-related gene expression in two TNBC cell lines (BT-549 and MDA-MB-21) and in a stabilized, primary patient-derived TNBC cell line (MS-186) compared to the STD schedule. Conventional high-dose schedules typically kill rapidly cycling cells, exerting insufficient pressure on slow-cycling CSCs, which then drive relapse. In contrast, the continuous, low-dose exposure characteristic of mCHT applies sustained anti-angiogenic and cytotoxic stress that successfully targets the survival mechanisms of the CSC population. These findings expand our previous work on mCHT in breast cancer, where we reported anti-angiogenic, immunomodulatory, and direct anti-tumor effects [8,9,10,19], and now provide evidence that mCHT also effectively targets the CSC compartment, a critical driver of TNBC progression and relapse. Our observations corroborate this mechanistic distinction.

First, we show that mCHT treatment of spheroids derived from commercial TNBC cell lines is more effective than STD schedule in significantly reducing their size (Figure 2). Remarkably, the effect is even more pronounced when analyzing 2nd-generation spheroids (Figure 3), which are highly enriched in self-renewing CSCs, thus suggesting that mCHT treatment likely impacts primarily on CSCs. Supporting this conclusion are data on mRNA (Figure 4) and protein expression (Figure 5) of key stemness markers such as CD44, CD133, NOTCH-1, and MYC. To note, not all genes are downregulated to the same extent in the two different cell lines, and some are also differentially modulated. We hypothesize that the different genetic backgrounds of the two cell lines may impact on the expression of the single stemness markers. MDA-MB-231 and BT-549 belong to distinct molecular subtypes of TNBC (e.g., Mesenchymal-like or Basal-like 2, respectively) and exhibit varied molecular dependencies, including differential activity of pathways such as the Androgen Receptor or Insulin-like Growth Factor signaling [20,21]. In addition, commercial cell lines usually have accumulated additional mutations, given the long time since they have been initially derived. However, the fact that, in general, mCHT decreased the expression of the stemness markers more efficiently that the STD administration and across these variable genetic backgrounds strengthens the finding that this schedule targets a fundamental and shared vulnerability across heterogeneous TNBC subsets. In addition, long-term mCHT treatment is required to efficiently suppress stemness traits, as indicated by the observation that the most significant reductions in expression are generally observed in 2nd-generation spheroids (Figure 5A,B). The discrepancy between the levels of the RNA and the relative protein observed in some cases could be due to post-transcriptional regulation and to a variety of epigenetic modifications as described in the literature [22]. Notably, some proteins are upregulated by STD treatment in the 1st-generation spheroids; for example, CD133 is upregulated in spheroids derived from both cell lines, MYC levels increase in BT-549-derived spheroids, whereas NOTCH-1 levels increase in MDA-MB-231-derived spheroids. It seems likely that the STD schedule might induce the expression of stemness-related genes to select drug-resistant cells.

Second, the immunofluorescence data obtained on cell line-derived spheroids confirm the disruptive effect of mCHT compared to STD schedule (Figure 6 and Figure 7). In fact, the signals from all stemness markers (CD133, NOTCH-1, MYC) are strongly reduced, if not abolished, and the overall architecture of the spheroid is severely compromised, as indicated by the loss of signal for the cytoskeletal protein F-actin.

Finally, a striking confirmation of the effect of mCHT on stemness traits has been obtained in a model closer to the patients, i.e., a stabilized primary cell line derived from a TNBC patient and grown in 3D. Here, the effect is strong already in 1st-generaton spheroids (Figure 8A). Accordingly, all the stemness markers are downregulated at the mRNA level (Figure 8B) by mCHT, whereas STD treatment induces the expression of stemness-related genes, suggesting that this kind of therapy may even be detrimental given that it may select resistant cells. Corroborating this suggestion is the data obtained on protein expression (Figure 9) where the transitory downregulation of stemness markers in STD-treated 1st-generation spheroids is not maintained in 2nd-generation spheroids (highly enriched in self-renewing CSCs), as all the proteins are upregulated. This phenomenon is known as Chemotherapy-Induced Cancer Stemness (CICS), where cytotoxic stress selects for pre-existing resistant cells or actively induces stemness characteristics in residual cells [23,24]. Notably, in mCHT-treated 2nd-generation spheroids, protein expression of MYC, NOTCH-1, CD44, and CD133 is remarkably downregulated. These results suggest that prolonged, low-dose exposure preferentially affects the most therapy-resistant subpopulation of tumor cells, functioning effectively as a CICS blocker and leading to sustained suppression of the tumor-initiating capacity. Mechanistically, this effect in spheroids derived from MS-186 cells was accompanied by a significant reduction in the expression of EZH2, a histone methyltransferase of the polycomb repressive complex 2 (PRC2) that has been implicated in breast cancer aggressiveness and poor prognosis in TNBC [25,26]. EZH2 overexpression is reported in up to 55% of invasive breast cancer and is strongly associated with ER-/PR- status, high histological grade, and TNBC phenotype [27]. Importantly, it has been shown that EZH2 promotes the expansion of breast stem cells through the transcriptional activation of NOTCH-1 signaling [28]. The concurrent downregulation of EZH2 and NOTCH-1 (Figure 9A,B) in our study supports this mechanistic link, suggesting that mCHT may disrupt a key pathway that maintains CSC self-renewal. These observations are in line with our previous clinical report, which demonstrated modulation of NOTCH-1 and its downstream targets (CD44, CD133, and MYC) in response to mCHT followed by conventional therapy in a patient with advanced TNBC [29]. Collectively, these data strengthen the hypothesis that mCHT can reprogram CSC-related signaling networks, resulting in long-term suppression of tumor-initiating capacity.

Genetic or pharmacological inhibition of EZH2 has been shown to reduce proliferation, delay G2/M transition, and induce a mesenchymal-to-epithelial transition, with E-cadherin restoration [30]. Importantly, growing evidence suggests that EZH2 may exert oncogenic functions beyond its histone methyltransferase activity, likely being involved in non-canonical protein–protein interactions that drive tumorigenesis [31]. This aspect is clinically relevant because many EZH2 inhibitors tested in clinical trials, such as the SAM-competitive EZH2 inhibitor tazemetostat (approved for advanced epithelioid sarcoma), primarily target the methyltransferase domain and may not fully abrogate these non-canonical oncogenic functions [32]. Alternative strategies, including EED-binding molecules (e.g., MAK683) [33,34] or EZH2-targeting PROTACs, which degrade EZH2 protein, have shown promise in preclinical and early clinical studies by degrading EZH2 protein and disrupting its broader oncogenic activity [35,36,37,38]. Based on our data, we put forward the hypothesis that the ability of mCHT to strongly reduce EZH2 protein levels could disrupt the physical scaffolding necessary for EZH2 to bind the NOTCH-1 promoter and drive transcription. This mechanism, together with sustained suppression of key stemness markers (CD44, CD3, NOTCH-1, MYC), ref. [29], may represent a central mechanism leading to the depletion of CSCs and the reduction in spheroid-forming capacity.

Notably, following mCHT treatment of MS-186-derived spheroids, we observed not only EZH2 downregulation but also suppression of both phosphorylated and total STAT3 (Figure 9B). STAT3 is a transcription factor activated by IL-6 and other cytokines, promoting the expansion of CD44+/CD24− stem-like cells [39,40]. The recent evidence from both preclinical and clinical studies has established that STAT3 plays a critical role in TNBC, and that STAT3 inhibitors have shown efficacy in inhibiting TNBC tumor growth and metastasis [41].

The interplay between EZH2 and STAT3 provides a second mechanism that mCHT appears to disrupt. EZH2 is implicated in non-histone methylation, a non-canonical function that allows it to regulate various transcription factors. In particular, EZH2 has been shown to methylate STAT3 at lysine 49 directly, enhancing its nuclear localization and transcriptional activity [42,43,44,45]. The simultaneous inhibition of NOTCH-1 and STAT3 via EZH2 observed in our study supports the existence of a cooperative regulatory network that maintains CSCs. By suppressing EZH2 protein expression, mCHT efficiently reduces the availability of EZH2 and blocks the sustained genetic control (NOTCH-1 axis) and non-histone modification (STAT3 axis). This multimodal attack probably minimizes the signaling redundancy typical of aggressive TNBC, achieving both rapid signaling blockade (suppressed phosphorylated STAT3) and sustained genetic/epigenetic silencing (downregulated EZH2/NOTCH-1).

Interestingly, in mCHT-treated 2nd-generation spheroids derived from MS-186 cells, we also observe a decrease in H3K27ac, a marker of active enhancers and promoters, associated with gene activation. When mCHT induces the physical depletion of the EZH2 protein scaffold, it disrupts the architectural foundation required for EZH2 to bind these active chromatin regions characterized by H3K27ac mark [16]. The resulting shutdown of this specific, EZH2-dependent oncogenic transcription (such as the NOTCH-1/MYC axis) leads to the erasure of the corresponding H3K27ac mark. Therefore, the observed reduction in H3K27ac is a powerful molecular indicator that mCHT has successfully terminated EZH2’s non-canonical role as an oncogenic driver. Although this finding may seem counterintuitive, it is consistent with the hypothesis that mCHT induces a global reprogramming of chromatin, leading to reduced transcription of oncogenic genes toward suppression of critical drivers. This observation requires further mechanistic investigation.

## 4. Materials and Methods

### 4.1. Two-Dimensional Cell Culture

Human TNBC cell lines MDA-MB-231 and BT-549 were purchased from American Type Culture Collection (LGC Standards, Sesto San Giovanni, Italy) and cultured in DMEM and RPMI-1640 medium, respectively (Lonza, Euroclone, Pero, Italy), supplemented with 10% FBS (#ECS5000L, Euroclone, Pero, Italy), 100 units/mL of penicillin and 100 mg/mL of streptomycin (#ECB3001_3380; Euroclone, Pero, Italy). In addition, RPMI-1640 medium was supplemented with 0.023 units/mL of insulin. TNBC primary cell line MS-186 was cultured in the MammoCult Human Medium Kit (Stemcell Technologies, Diatech Lab Line srl, Jesi, Italy) supplemented with heparin solution (4 µg/mL) and hydrocortisone (0.48 µg/mL). Cells were routinely tested for Mycoplasma by Hoechst stain (#62249; Thermo Fisher, Monza, Italy). All cells were kept in a humidified incubator with 5% CO_2_ at 37 °C.

### 4.2. Spheroid Formation

IC_50_ values for 5-FU and VNR were previously determined for TNBC cell lines under both standard STD and mCHT regimens, as reported in our prior work; for MS-186 cells, IC_50_ values were determined by using the MTT assay, as previously described [8]. A combination of IC_50_ 5-FU + IC_50_ VNR was then used to treat each cell line for 4 h (STD) or for 96 h (mCHT), changing the medium every 24 h. Control cultures were supplemented with vehicle alone (DMSO) and indicated throughout the paper as “not treated” (NT). Concentrations of drugs provoking 50% cell growth inhibition (IC_50_) were calculated from curves derived by plotting cell viability (%) versus drug concentration (nM). The reading values were converted to the percentage of the control.

STD treatment with IC_50_ 5-FU+IC_50_ VNR was calculated as 80,000 nM 5-FU + 30 nM VNR and 100,000 nM 5-FU + 35 nM VNR, for MDA-MB-231 and BT-549 cells, respectively. IC_50_ 5-FU + IC_50_ VNR for mCHT treatment were 4500 nM 5-FU + 0.50 nM VNR for both cell lines. STD treatment with IC_50_ 5-FU + IC_50_ VNR for MS-186 cells was determined as 312,500 nM 5-FU + 30 nM VNR, whereas for mCHT treatment, the IC_50_ was 13,000 nM 5-FU + 2 nM VNR. At the end of the treatment period, TNBC cells were counted, and 50,000 viable cells/well were seeded into 6-well ultra-low attachment plates in complete medium supplemented with 2% Matrigel (Thermo Fisher, Monza, Italy) to promote 3D spheroid formation. MS-186 cells were seeded into 6-well ultra-low with appropriate medium *w*/*o* Matrigel. Primary spheroids (1st generation) were typically formed in 72 h. After formation, spheroids were cultured for an additional 7 days to allow maturation. After this period, spheroids were collected and disaggregated mechanically using a sterile needle. The resulting single cells were reseeded under the same conditions (*w*/*o* treatments) to generate 2nd generation of spheroids, which were grown for 10 days. Both 1st- and 2nd- generation spheroids were processed for different purposes, as depicted in Figure 1. Mainly, they were pelleted and stored at −80 °C for RNA and protein extraction or fixed for fluorescence staining.

### 4.3. RNA Extraction and Real-Time PCR

Total RNA from TNBC cell lines was isolated by RNeasy Mini Kit (QIAGEN srl, Milano, Italy) according to the manufacturer’s instructions. RNA quantity and quality were evaluated with a Nanodrop ND2000 spectrophotometer (Thermo Fisher Scientific, Monza, Italy). After measuring the concentrations, total RNA was reverse-transcribed using the High-Capacity cDNA Reverse Transcription Kit (Thermo Fisher Scientific, Monza, Italy). For each sample, 1 µg of RNA was reverse-transcribed. Real-time PCR was performed using TaqMan Gene Expression Master Mix (Thermo Fisher Scientific, Monza, Italy), and the plates were analyzed by thermocycler StepOnePlus Real-time PCR System (Thermo Fisher Scientific, Monza, Italy). All TaqMan probes, CD133 (Hs00608272_m1), CD44 (Hs01075861_m1), NOTCH-1 (Hs01062014_m1), MYC (Hs00153408_m1) and GAPDH (Hs99999905_m1) were purchased from Life Technologies (Thermo Fisher Scientific, Monza, Italy). Expression levels of stemness-related genes (CD44, CD133, NOTCH-1, and MYC) were normalized to GAPDH and reported as relative fold changes. Three separate experiments were performed, and values were subjected to statistical analysis and plotted graphically.

### 4.4. Western Blot Analyses

MDA-MB-231, BT-549, and MS-186 cells were treated with the respective IC_50_ of 5-FU+VNR under the mCHT or STD schedule and lysed with RIPA buffer (HEPES 50 mM, pH 7.5, NaCl 500 mM, DTT 1 mM, EDTA 1 mM, 0.1% NP40) supplemented with 1% protease inhibitor cocktail (# P2714, Sigma-Aldrich, Milan, Italy). Protein concentration was measured by the Bradford method (# B6916, Sigma-Aldrich, Milan, Italy). An amount of 20 μg proteins were loaded into 10% NuPAGE Tris-Glycine protein gels or 4–12% NuPAGE Bis Tris protein gels (Thermo Fisher, Monza, Italy) and run for 2 h at 100 V in Tris-Glycine Running buffer or MES Running buffer (Thermo Fisher, Monza, Italy). Proteins were transferred to nitrocellulose membrane (Thermo Fisher, Monza, Italy) by the iBlot system, which were blocked for 1 h in a 0.5% BSA or milk solution and then incubated overnight at 4 °C with the following primary antibodies: anti-Ab CD44 and anti-CD133 (#189524 and #ab216323, respectively, Abcam, Prodotti Gianni, Milano, Italy), anti-MYC and anti-NOTCH-1 (D84C12 Rabbit mAb **#**5605 and D6F11 XP^®^ Rabbit mAb **#**4380, respectively, Cell Signaling, Euroclone, Pero, Italy). Blots were washed twice with 0.05% Tween-TBS and probed with HRP-conjugated donkey anti-rabbit or goat anti-mouse Ig (Cell Signaling, Euroclone, Pero, Italy) for 1 h at room temperature. Protein loading was confirmed by probing the blots with HRP-conjugated rabbit anti-actin Ab (#12620, Cell Signaling, Euroclone, Pero, Italy). After three washes with 0.05% PBS-Tween, membranes were incubated with Pierce™ ECL Western Blotting Substrate (#32106, Thermo-Fisher, Monza, Italy) for 5 min and proteins were detected using G:BOX Chemi System device (SynGene; Cambridge, UK). GAPDH was used as a loading control and to perform densitometric evaluation of relative protein levels by use of the ImageJ software (https://imagej.net/ij/ version 1.53, downloaded 21 July 2021. Three separate experiments were performed, and values were subjected to statistical analysis and plotted graphically.

### 4.5. Immunofluorescence

For immunofluorescence assays, BT-549, MDA-MB-231, and MS-186 cells were treated with the respective IC_50_s of 5-FU+VNR under either the mCHT or STD schedule. At the end of treatment, cells were counted, and 3000 cells were seeded into 96 u-well plates. For BT-549 and MDA-MB-231, a base layer of 1.5% agarose was prepared by dissolving it in 50 μL of milliQ H_2_O boiling until fully dissolved, then mixed with 50 μL of appropriate medium per well before adding cells. MS-186 cells were plated without agarose. Spheroids of the 1st and 2nd generations were collected at respective time points and transferred into 1.5 mL Eppendorf tubes (Euroclone, Pero, Italy). After centrifugation at 2000 rpm for 10 min, the samples were washed in PBS and fixed with 4% paraformaldehyde for 20 min. After fixation, samples were washed three times with PBS and incubated in blocking solution (1X PBS, 5% normal serum, and 0.3% Triton X-100) for 1 h. After PBS washes, samples were incubated with primary antibody anti-CD44 and anti-CD133 (#189524 and #ab216323, respectively, Abcam, Prodotti Gianni, Milano, Italy), anti-MYC and anti-NOTCH-1 (D84C12 Rabbit mAb **#**5605 and D6F11 XP^®^ Rabbit mAb **#**4380, Cell Signaling, Euroclone, Pero, Italy) in PBS containing 1% BSA and 0.3% Triton X-100 overnight on a tube rotator. Samples were subsequently washed and incubated with specific secondary antibody conjugated with Alexa 488 (Thermo Fisher, Monza, Italy) in 1% BSA in PBS with 0.3% Triton X-100, along with DAPI and Phalloidin-Alexa Fluor 568 conjugated probes (#A12380, Molecular Probes, Thermo Fisher, Monza, Italy). This incubation lasted 2 h on the rotator, followed by two washes in PBS. After removal of the excess liquid, mounting medium was added to a chamber slide.

Images were acquired using a Zeiss LSM 710 confocal laser-scanning microscope (Carl Zeiss S.p.A., Milano, Italy) using a 25×, 0.8 N/A oil-immersion objective. Laser intensities and acquisition parameters were held constant throughout each experiment. Confocal microscopy fields were analyzed using specific homemade-designed macro with ImageJ software. In detail, signal intensities were analyzed, measuring the ID normalized over the signal obtained in the control growing condition. All the data obtained were derived from at least 15 different z planes per experimental condition.

### 4.6. Statistical Analysis

All experiments were performed three times and values resulting from quantification of spheroid area, spheroids counting, RT-PCR fold changes, and densitometric analysis of Western blotting were subjected to two-tailed *T* test. All graphs are plotted using the mean values ± SEM and asterisks used to indicate *p* < 0.05 between two groups of treatment as indicated in the legend.

## 5. Conclusions

The presented data indicate that mCHT 5-FU+VNR reprograms CSCs in TNBC spheroids mainly by suppressing the EZH2/NOTCH-1/STAT3 axis; notably, blockade of these pathways represents a novel anti-stem cell mechanism that synergizes with the established multi-targeted actions of mCHT (anti-angiogenesis, induction of apoptosis, and inhibition of proliferation). In fact, these regulators are essential for maintaining and self-renewing CSCs, enabling resistance to therapy, and promoting metastatic potential; thus, their downregulation aligns with the observed reduction in spheroid growth and the impaired formation of 2nd-generation spheroids under metronomic 5-FU+VNR treatment. The functional benefits of this multimodal suppression provide a clear therapeutic advantage by preventing relapse through the elimination of the self-renewing CSC population—the primary driver of tumor recurrence and metastasis. Notably, mCHT offers the potential for more long-lasting responses, preventing the rebound of CD44, CD133, cl-NOTCH-1, and MYC as noted, in particular, under STD treatment of MS-186 primary cell line-derived spheroids. The suppression of these markers is directly linked to the schedule of treatments, which actively prevents the selection and induction of drug-resistant stemness traits (CICS), a significant limitation of conventional therapy. Overall, these results support mCHT as a strategy for a more durable therapeutic response by targeting CSC-driven recurrence.

Further in vivo studies will better clarify CSC inhibition and assess whether combining mCHT with EZH2- or STAT3-targeted agents could improve long-term tumor control. Additional mechanistic analyses, including ChIP-seq and proteomics, are needed to define the non-canonical EZH2 pathways affected by mCHT. We acknowledge the limitations of the present study—namely, the lack of FACS-based protein quantification of protein expressed on the surface of the spheroids and the absence of an in vivo model—which will be addressed in forthcoming work. Nonetheless, the current data provide a solid basis for the above-mentioned investigations.

## Figures and Tables

**Table 1:** IC_50_ values for 5-FU and VNR under STD or mCHT conditions in MS-186 cells.

		Single Treatment		Combo Treatment	
		Treatment	IC_50_ ^(a)^	Treatment	IC_50_ ^(a)^
**MS-186**	**STD**	**5-FU**	625,000 nM	**5-FU+VNR**	312,500 nM + 30 nM
		**VNR**	60 nM		
	**mCHT**	**5-FU**	26,000 nM	**5-FU+VNR**	13,000 nM + 2 nM
		**VNR**	4 nM		

^(a)^ IC_50_ values were calculated from dose–response curves based on the cell viability assay and represent the concentration of each drug or 5FU+VNR combination required to achieve 50% inhibition of cell viability.

## Data Availability

The original contributions presented in this study are included in the article. Further inquiries can be directed to the corresponding authors.

## Abbreviations

The following abbreviations are used in this manuscript:

TNBC	Triple Negative Breast Cancer
mCHT	Metronomic Chemotherapy
STD	Standard Treatment
5-FU	5-fluorouracil
VNR	Vinorelbine
SCS	Cancer Stem Cells
NT	Not Treated
